# Recombinant human thrombomodulin attenuated sepsis severity in a non-surgical preterm mouse model

**DOI:** 10.1038/s41598-019-57265-2

**Published:** 2020-01-15

**Authors:** Mariko Ashina, Kazumichi Fujioka, Kosuke Nishida, Saki Okubo, Toshihiko Ikuta, Masakazu Shinohara, Kazumoto Iijima

**Affiliations:** 1 Department of Pediatrics, Kobe University Graduate School of Medicine, Kobe, Japan; 2 Division of Epidemiology, Kobe University Graduate School of Medicine, Kobe, Japan

**Keywords:** Lipidomics, Paediatric research, Antimicrobial responses

## Abstract

Neonatal sepsis is characterised by dysregulated immune responses. Lipid mediators (LMs) are involved in the regulation of inflammation. Human recombinant thrombomodulin (rhTM), an anticoagulant, has anti-inflammatory effects and might be useful for sepsis treatment. A stock caecal slurry (CS) solution was prepared from adult caeca. To induce sepsis, 1.5 mg/g of CS was administered intraperitoneally to 4 d-old wild-type FVB mouse pups. Saline (Veh-CS) or rhTM (3 or 10 mg/kg; rhTM3-CS or rhTM10-CS) was administered subcutaneously 6 h prior to sepsis induction, and liver LM profiles at 3 and 6 h post-sepsis induction and survival up to 7 days were examined. Mortality was significantly lower (47%) in the rhTM3-CS group and significantly higher (100%) in the rhTM10-CS group, compared with the Veh-CS group (79%, p < 0.05). Eleven LMs (12-HEPE, EPA, 14-HDHA, DHA, PD1, PGD_2_, 15d-PGJ_2_, 12S-HHT, lipoxin B_4_, 12-HETE, AA) were significantly increased at 3 h, and five LMs (5-HEPE, 15-HEPE, 18-HEPE, 17-HDHA, PD1) were significantly increased at 6 h post-sepsis induction. Increased EPA, DHA, 12S-HHT, lipoxin B_4_, and AA were significantly suppressed by rhTM pre-treatment. rhTM was protective against neonatal sepsis. This protective effect might be mediated via LM modulation. Further post-sepsis studies are needed to determine clinical plausibility.

## Introduction

Neonatal sepsis is characterised by systemic bacterial invasion followed by a massive inflammatory response. The mortality rate of neonatal sepsis is variable and was reported to be ≥35%^1^. Of note, the mortality rate of sepsis in preterm infants is two-fold higher than that in term infants. In addition, preterm sepsis was reported to be associated with adverse neurodevelopmental outcomes, such as cerebral palsy, hearing impairment, and neurodevelopmental impairment^1^. Although the pathophysiology of neonatal sepsis is not fully elucidated, altered premature immune responses have been hypothesised to play a crucial role^2,3^. In addition to the well-characterised functions of inflammatory cytokines and chemokines, bioactive lipid mediators (LMs) were recently discovered to be key signalling molecules that regulate the inflammatory profile^4^. LMs are biosynthesised through specific metabolic pathways and exert various bioactive effects through their specific receptors, which have two opposite functions: inflammatory and anti-inflammatory^5,6^. The anti-inflammatory function of LMs is reportedly associated with the resolution of inflammation, and the dysregulated balance of LMs has attracted attention as a pathway involved in the pathogenesis of sepsis^4^.

Currently, there are guidelines for the treatment of sepsis in adults and children, including term newborns; however, there are no established guidelines for the treatment of sepsis in preterm infants^1^. Single antibiotic therapy for sepsis in very low birth weight infants is ineffective, and adjunctive immunotherapy, such as granulocyte stimulating factor and immunoglobulin treatment, did not improve the long-term outcome of preterm sepsis^7^. In adult sepsis, novel therapeutic options such as interleukin-22, anti-high mobility group box 1 (HMGB1) monoclonal antibody, vascular endothelial growth factor receptor monoclonal antibodies, and recombinant human thrombomodulin (rhTM) are the focus of preclinical investigation^8^. Furthermore, low-dose corticosteroids^9^, orally administered protease inhibitors^10^, and mesenchymal stem cell therapy^11^ are currently being investigated in clinical trials. However, no such trials have been initiated for neonatal sepsis.

The biological agent rhTM was approved and is used clinically for disseminated intravascular coagulation (DIC) treatment in Japan^12,13^. The effects of rhTM on DIC were previously established in a multicentre randomised controlled trial^14^, which showed that the beneficial effect was associated with a reduction in mortality in adult sepsis patients with DIC^12^. To exert its anticoagulant effect, rhTM forms a complex with thrombin to inhibit clotting activity, and the thrombin-rhTM complex itself converts protein C (PC) to activated protein C (APC), which selectively inactivates activated factor V (Va) or activated factor VIII (VIIIa). This results in the inhibition of further thrombin formation^15^. Recently, the pleiotropic and anti-inflammatory effects of rhTM have attracted attention. These include inhibition of intercellular adhesion molecule-1 expression (ICAM-1), activation of protease activated receptor-1 (PAR-1)^15^, neutralisation of endotoxins, and adsorptive dissolution of HMGB-1 proteins^16^. Because rhTM has anti-coagulative and anti-inflammatory effects, it is regarded as a useful therapeutic agent for sepsis. Importantly, Shirahata *et al*. reported post-marketing surveillance data regarding the use of rhTM in neonatal DIC in Japan; these data showed that its safety and effectiveness were similar to those in paediatric and adult patients with DIC^17^.

However, the efficacy of rhTM for the treatment of sepsis in preterm infants has not been determined. In addition, although the target of LMs is presumed to be the pathology of sepsis, there has been no human or animal study on the effects of LMs in neonatal sepsis.

Therefore, to clarify the effect of rhTM on sepsis in preterm infants, we investigated the protective effect of subcutaneous rhTM administration in a non-surgical preterm sepsis mouse model, and investigated the dynamics of LMs in this model.

## Methods

### Animals

Adult FVB/NJcl mouse breeders were obtained from CLEA Japan, Inc. (Tokyo, Japan) and provided a standard rodent diet and water *ad libitum*. All pups were kept with their mothers throughout the course of the study. The pups were randomised on an individual basis within each litter for each experiment. At least three different litters were used for each experimental group to eliminate any litter bias effects. For identification, the back of each pup was labelled using a Sharpie marker. This study was carried out in accordance with the ARRIVE guidelines and performed as approved by the Kobe University Institutional Animal Care and Use Committee (Protocol P160608).

### Bacterial viability of the caecal slurry (CS) stock

As described previously^2^, a single stock CS solution was prepared from adult caeca, and then stored at −80 °C in 1-mL aliquots until use. At each sepsis induction, an aliquot of stock CS was thawed at room temperature, and 50 µl was plated onto 1.5% agar containing brain/heart infusion (BHI) broth^18^. Agar plates were then incubated at 37 °C for 24 h and colony-forming units (CFUs) were counted. For the entire study, the mean CFU count was 5.2 ± 1.2 × 10^5^ CFU/mL.

### Sepsis induction

To induce sepsis, 4-day-old mouse pups, which are immunologically equivalent to human preterm infants^2,3^, were given various doses of CS intraperitoneally (IP), and then closely monitored daily for health and survival for up to 7 days, based on the method used in a previous report^18^. For the following experiment, we used a CS dose of LD80 for sepsis induction, based on the approach in our previous studies^2,3^.

### Recombinant human thrombomodulin (rhTM) treatment

To determine the safety of rhTM in neonatal pups, we administered 10.0 mg/kg of rhTM subcutaneously (SC) to 4-day-old mice and monitored survival for 7 days. Injection of 10.0 mg/kg of rhTM SC to 4-day-old mice did not induce mortality for up to 7 days (100% survival, n = 10). At 6 h before-sepsis induction, we administered 3.0 mg/kg of rhTM (rhTM3-CS), 10.0 mg/kg of rhTM (rhTM10-CS), or an equivalent volume of normal saline (Veh-CS) SC to 4-day-old mice, based on the method used in a previous report^19^. The timing of rhTM SC administration was determined based on a pharmacokinetic study to achieve a peak concentration at sepsis induction. In a pharmacokinetic study of adult rats, a single subcutaneous injection of 3.0 mg/kg of rhTM increased blood concentration slowly, such that it reached sufficient levels between 3 and 9 hours after administration^20^. At 6 h after rhTM administration, sepsis was induced by IP administration of 1.5 mg/g body weight (BW) CS; BW changes and survival were then monitored for 7 days. Blood gas and liver LM analysis was performed at 3 and 6 h post-sepsis induction based on the methods used in previous reports^21,22^.

### Blood gas parameters

At 3 h post-sepsis induction, pups were sacrificed by decapitation under room air and 30 to 80 µL of blood pooled from two or three pups was immediately collected in capillary blood collection tubes containing lithium heparin (Capiject®; Terumo Medical Corporation, Tokyo, Japan). All measurements were performed using an ABL 90 FLEX blood gas analyser (Radiometer Medical ApS, Copenhagen, Denmark).

### Measurement of lipid mediators

At 3 and 6 h post-sepsis induction, pups were sacrificed and approximately 5 × 5 × 1 mm pieces from freshly harvested livers were placed in liquid nitrogen and then stored at −80 °C until use. The liver samples were homogenised in ice-cold methanol. Then, the deuterated internal standards d_4_-leukotriene B_4_ (d_4_-LTB_4_), d_8_-5-hydroxyeicosatetraenoic acid (d_8_-5-HETE), d_4_-prostaglandin E_2_ (d_4_-PGE_2_), and d_5_-resolvin D2 (d_5_-RvD2), which represented each chromatographic region of the identified LMs, were added to the samples (500 pg each) to facilitate quantification. Samples underwent solid phase extraction (SPE) on C18 columns and were subjected to LC-MS/MS. The system consisted of a Qtrap 6500 (Sciex, Framingham, MA, USA) equipped with a Shimadzu LC-30AD HPLC system. A ZORBAX Eclipse Plus C18 column (100 mm × 4.6 mm, 3.5 µm, Agilent Technologies, Santa Clara, CA, USA) was used with a gradient of methanol/water/acetic acid from 55:45:0.01 (v/v/v) to 98:2:0.01 at a flow rate of 0.4 ml/min. A multiple reaction monitoring (MRM) method was developed for the signature ion pairs Q1 (parent ion)/Q3 (characteristic fragment ion) of each molecule to monitor and quantify the levels of targeted LMs. The LMs were identified using published criteria, including LC retention time, specific fragmentation patterns, and diagnostic fragmentation ions. The samples were quantified based on the peak area of the MRM chromatograph, and linear calibration curves were obtained with authentic standards for each compound^5,23^.

### Statistical analyses

Statistical analyses were performed using log-rank tests for Kaplan–Meier survival curves, unpaired Student’s two-tailed *t*-test, Mann–Whitney *U-*test or Chi-square test for comparisons between two groups. Statistical analyses were performed using GraphPad Prism 7.0 software (Graphpad Software, Inc., San Diego, CA, USA). Differences were deemed statistically significant when p < 0.05.

## Results

### Effect of rhTM on the severity of sepsis

We randomly assigned mouse pups into four groups: non-septic control (vehicle [Veh]-pretreated normal saline IP administration, Veh-Veh) group, Veh-treated septic (Veh-CS) group, and rhTM pretreated septic (rhTM3-CS or rhTM10-CS) groups, based on the method used in a prior study^24^.

#### BW Change

**Figure 1:**
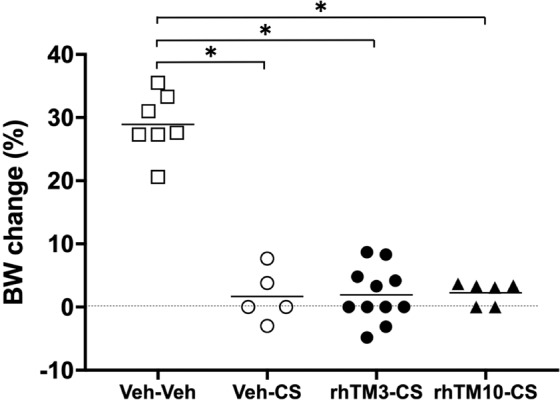
Body weight gain at 24 h post-sepsis induction. Body weight gain of surviving pups only is shown: vehicle-treated septic (Veh-CS) group (1.7 ± 4.1%, n = 5), 3.0 mg/kg of rhTM pretreated septic (rhTM3-CS) group (1.9 ± 4.3%, n = 11), 10.0 mg/kg of rhTM pretreated septic (rhTM10-CS) group (2.3 ± 1.8%, n = 6), and non-septic control (Veh-Veh) group (28.9 ± 4.9%, n = 7). The body weight changes of Veh-CS, rhTM3-CS, and rhTM10-CS groups were significantly smaller than the body weight change of the Veh-Veh group. *p < 0.0001.

When comparing the BW gain at 24 h post-sepsis induction of surviving pups only, there were no significant differences among the Veh-CS (1.7 ± 4.1%, n = 5), rhTM3-CS (1.9 ± 4.3%, n = 11), and rhTM10-CS (2.3 ± 1.8%, n = 6) groups. However, the BW gain of these groups was significantly lower than the BW gain of the non-septic Veh-Veh group (28.9 ± 4.9%, n = 7, p < 0.0001, Fig. 1).

#### Blood gas

At 3 h post-sepsis induction, lactate levels, anion gaps, and blood glucose were significantly higher in pups of the Veh-CS group than in the Veh-Veh controls (p < 0.05, Table 1). However, blood glucose was significantly higher and blood pH indicated significantly lower alkalinity in the rhTM3-CS and rhTM10-CS groups, compared with the Veh-Veh controls (p < 0.05, Table 1). In addition, lactate levels and anion gaps were significantly lower and pCO_2_ was significantly higher in the rhTM10-CS group than in the Veh-CS group (p < 0.05, Table 1). There were no significant differences in blood gas parameters between the rhTM3-CS and rhTM10-CS groups.

**Table 1:** Effects of rhTM on blood gas parameters.

	Veh-Veh (n = 7)	Veh-CS (n = 5)	rhTM3-CS (n = 6)	rhTM10-CS (n = 4)
pH	7.57 ± 0.09	7.48 ± 0.03	7.46 ± 0.02^*^	7.46 ± 0.04^*^
pO_2_ (mm Hg)	164.5 ± 29.6	175.0 ± 18.3	173.2 ± 17.5	162.1 ± 24.3
pCO_2_ (mm Hg)	25.8 ± 4.3	25.5 ± 2.3	29.5 ± 4.3	30.5 ± 2.9^†^
AG (meq/L)	9.7 ± 1.6	12.3 ± 1.8^*^	10.9 ± 2.3	8.6 ± 2.3^†^
Lac (mmol/L)	5.9 ± 2.9	9.5 ± 1.0^*^	8.4 ± 0.6	7.8 ± 0.9^†^
Glucose	77.3 ± 38.2	158.4 ± 38.4^*^	160.0 ± 46.9^*^	151.0 ± 19.4^*^

Results are expressed as mean ± SD. *p < 0.05 vs Veh-Veh, ^†^p < 0.05 vs Veh-CS.

#### Mortality

**Figure 2:**
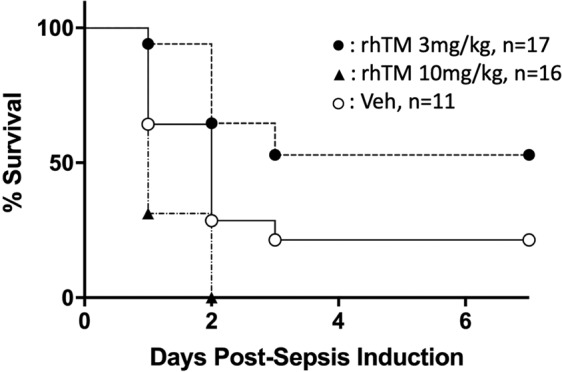
Kaplan–Meier survival plots of 4 d-old pups pretreated with 3.0 mg/kg of rhTM (rhTM3-CS: ●, n = 17), 10.0 mg/kg of rhTM (rhTM10-CS: ▲, n = 16), or equivalent volume of normal saline (Veh-CS: ○, n = 11) subcutaneously at 6 h pre-sepsis induction. The mortality was significantly lower in the rhTM3-CS group (47%) and significantly higher in the rhTM10-CS group (100%), compared with the Veh-CS group (79%).

The mortality rates of Veh-CS-treated pups (79%, n = 11), rhTM3-CS-treated pups (47%, n = 17), and rhTM10-CS-treated pups (100%, n = 16) were significantly higher than the mortality rate of Veh-Veh-treated pups (0%, n = 7, p = 0.003, p = 0.04, and p < 0.0001, respectively). In addition, mortality was significantly lower in the rhTM3-CS group than in the Veh-CS-treated group (p = 0.03, Fig. 2). Conversely, mortality was significantly higher in the rhTM10-CS-treated group than in the Veh-CS-treated group (p = 0.02, Fig. 2).

### Effect of rhTM on lipid mediators

While the administration of 3.0 mg/kg rhTM resulted in improved survival, injection of 10.0 mg/kg did not show a protective effect; thus, the following studies were conducted using only 3.0 mg/kg rhTM. At 3 h post-sepsis induction, the livers of Veh-CS-treated pups contained two eicosapentaenoic acid (EPA)-derived LMs (12-HEPE, EPA), three docosahexaenoic acid (DHA)-derived LMs (14-HDHA, DHA, PD1), and six arachidonic acid (AA)-derived LMs (PGD_2_, 15 d-PGJ_2_, 12S-HHT, lipoxin B_4_, 12-HETE, AA), which were significantly increased compared with the Veh-Veh-treated group. At 6 h post-sepsis induction, three EPA-derived LMs (5-HEPE, 15-HEPE, 18-HEPE) and two DHA-derived LMs (17-HDHA, PD1) were significantly increased in livers of Veh-CS-treated pups, compared with Veh-Veh-treated pups (Table 2, Supplementary Table S1).

**Table 2:** Effects of rhTM on LM parameters.

LM (pg/mg tissue)		Non-septic control	3 h post-sepsis induction		6 h post-sepsis induction	
		Veh-Veh (n = 4)	Veh-CS (n = 5)	rhTM3-CS (n = 6)	Veh-CS (n = 5)	rhTM3-CS (n = 5)
EPA	5-HEPE	0.7 ± 0.2	0.9 ± 0.2	0.9 ± 0.2	2.0 ± 0.4^*^	2.7 ± 1.3
	12-HEPE	3.1 ± 2.0	37.1 ± 12.2^*^	11.2 ± 3.8	17.4 ± 5.3	11.2 ± 3.1
	15-HEPE	0.6 ± 0.2	1.1 ± 0.3	0.8 ± 0.1	1.8 ± 0.4^*^	2.2 ± 0.8
	18-HEPE	1.0 ± 0.4	2.5 ± 0.8	1.8 ± 10.2	3.6 ± 0.7^*^	3.8 ± 1.4
	EPA	35.2 ± 15.0	200.9 ± 28.2^*^	80.1 ± 34.4^†^	98.6 ± 23.4	301.6 ± 41.6^*§^
DHA	14-HDHA	2.5 ± 0.9	17.7 ± 5.0^*^	7.6 ± 2.4	8.7 ± 2.3	9.4 ± 2.2^*^
	17-HDHA	2.9 ± 1.1	7.6 ± 2.5	5.0 ± 1.2	9.1 ± 2.0^*^	7.1 ± 2.4
	DHA	118.0 ± 43.0	903.4 ± 150.5^*^	351.8 ± 160.1^†^	442.9 ± 142.1	671.7 ± 110.4^*^
	PD1	0.0 ± 0.0	0.1 ± 0.0^*^	0.2 ± 0.0^*^	0.1 ± 0.0^*^	0.1 ± 0.0^*^
AA	PGD_2_	290.4 ± 82.6	832.2 ± 150.6^*^	648.7 ± 41.5^*^	487.6 ± 89.6	314.5 ± 40.6
	AA	106.6 ± 40.7	571.3 ± 89.8^*^	218.8 ± 75.7^*†^	384.7 ± 109.2	498.8 ± 66.2^*^
	15deoxy-d12,14 PGJ_2_	74.8 ± 20.6	224.5 ± 31.9^*^	173.2 ± 19.7^*^	107.9 ± 27.0	108.2 ± 22.0
	PGF_2a_	68.1 ± 25.7	237.8 ± 73.4	166.4 ± 15.0^*^	187.0 ± 46.2	180.6 ± 21.2^*^
	12S-HHT	6.4 ± 2.2	34.8 ± 9.0^*^	13.7 ± 1.8^*†^	27.1 ± 8.4	30.2 ± 7.4^*^
	Lipoxin B_4_	0.4 ± 0.1	2.9 ± 0.5^*^	1.2 ± 0.3^†^	0.7 ± 0.2	0.6 ± 0.1
	12-HETE	13.5 ± 6.9	166.0 ± 53.5^*^	54.4 ± 18.4	13.5 ± 3.7	11.1 ± 4.0

Results are expressed as mean ± SEM. *p < 0.05 vs control, ^†^p < 0.05 vs 3 h post-Veh-CS, ^§^p < 0.05 vs 6 h post-Veh-CS.

The significant increases of these LMs, including EPA, DHA, 12S-HHT, lipoxin B_4_, and AA, at 3 h post-sepsis induction were significantly suppressed by rhTM pre-treatment. Moreover, only EPA was significantly increased at 6 h post-sepsis induction in pups that received rhTM pre-treatment (Table 2, Supplementary Table S1).

#### Changes of LM levels compared with baseline

The change in LM level from baseline (non-septic control) was abbreviated as *Δ* LMs. The levels of *Δ* EPA, *Δ* DHA, *Δ* AA, *Δ* 12S-HHT, and *Δ* lipoxin B_4_ were significantly lower in the rhTM pre-treatment group than in the Veh-CS-treated group at 3 h post-sepsis induction. However, *Δ* EPA in the rhTM pre-treatment group was significantly higher than that in the Veh-CS-treated group at 6 h post-sepsis induction (Table 3, Supplementary Table S2).

**Table 3:** Effects of rhTM on *Δ*LM parameters.

*Δ* LM (pg/mg tissue)		3 h post-sepsis induction		6 h post-sepsis induction	
		Veh-CS (n = 5)	rhTM3-CS (n = 6)	Veh-CS (n = 5)	rhTM3-CS (n = 5)
EPA	EPA	165.7 ± 28.2	44.9 ± 34.4^*^	63.5 ± 23.4	266.4 ± 41.6^†^
DHA	DHA	785.3 ± 150.5	233.0 ± 160.1^*^	324.9 ± 142.1	553.7 ± 110.4
AA	AA	464.7 ± 89.8	112.2 ± 75.7^*^	278.1 ± 109.2	392.2 ± 66.2
	12S-HHT	28.4 ± 9.0	7.3 ± 1.8^*^	20.7 ± 8.3	23.8 ± 7.4
	Lipoxin B_4_	2.5 ± 0.5	0.8 ± 0.3^*^	0.3 ± 0.2	0.2 0.1

Changes from baseline (non-septic control) levels are abbreviated as *Δ* LM. Results are expressed as mean ± SEM. *p < 0.05 vs Veh-CS at 3 h post-sepsis induction, ^†^p < 0.05 vs Veh-CS at 6 h post-sepsis induction.

## Discussion

In this study, there were two important findings. First, subcutaneous rhTM administration attenuated sepsis severity in a non-surgical preterm sepsis mouse model. Second, levels of several inflammatory LMs were increased at 3 and 6 h post-sepsis induction, and these increases were partially suppressed by rhTM pre-treatment.

In our preterm sepsis mouse model, subcutaneous administration of 3 mg/kg of rhTM at 6 h prior to sepsis induction led to improvements in the blood gas parameters and mortality rate. In contrast, subcutaneous administration of 10 mg/kg of rhTM worsened the overall mortality, despite significant improvement of blood gas parameters at 3 h post-sepsis induction. To date, there have been several reports investigating the effect of rhTM in septic animal models. Nagato *et al*. reported that the intravenous administration of 1 mg/kg of rhTM 30 min prior to sepsis induction significantly improved sepsis survival, and suppressed inflammatory cytokines and HMGB1 elevation in an LPS-treated rat sepsis model^16^. Takehara *et al*. reported that the intravenous administration of 3 mg/kg of rhTM 30 min prior to sepsis induction improved sepsis survival, and suppressed the elevation of inflammatory cytokines and HMGB1 in serum and ascites in an LPS-treated mouse sepsis model^25^. However, these studies used adult rodent sepsis models, and there has been no report investigating the effects of rhTM in an animal model of neonatal sepsis. In addition, these studies utilised models of sepsis induced by LPS administration; however, sepsis induction using this model might not truly reflect the pathophysiology of human sepsis because LPS induces endotoxemia or systemic inflammation, but does not induce sepsis^26^. Furthermore, in LPS models, activation of the innate immune system can only have deleterious effects, whereby any intervention that blunts the inflammatory response can be beneficial; in contrast, sepsis in human patients is triggered by an infectious process in which immunological responses can be both beneficial and deleterious^27^. Thus, the caecal ligation and puncture model has been widely used because it closely resembles the progression and characteristics of human sepsis; however, newborn pups do not tolerate this surgically invasive procedure^28^. Here, we established a mouse model of non-surgical preterm sepsis using the CS method established by Wynn *et al*.^29^ and CS stock preparation protocol established by Starr *et al*.^18^. This is a simple technique consisting of intraperitoneal CS administration to 4-day-old mouse pups, an age immunologically equivalent to human preterm infants^30^. Similar to the caecal ligation and puncture model, the advantages of this model are the existence of an infection focus (i.e., an abdominal abscess) and its polymicrobial nature.

Regarding the dynamics of post-sepsis LMs, two EPA-, three DHA-, and six AA-derived LMs were increased 3 h post-sepsis induction, and six EPA- and two DHA-derived LMs were increased 6 h post-sepsis induction. Recently, the roles of LMs in maintenance of inflammation and their convergence have been reported in various inflammatory diseases. LMs exert various bioactive effects through their specific receptors, and are categorised as inflammatory LMs and anti-inflammatory LMs, which have diametrically opposite effects. Anti-inflammatory LMs are regarded as important pro-resolution mediators. The protective effect of anti-inflammatory LM administration was reported in an adult sepsis model mice^31,32^. Regarding LM dynamics in adult sepsis patients, Dalli *et al*. showed that inflammatory LMs, such as PGF_2α_, and anti-inflammatory LMs, including RvD5 and PD1, were significantly increased in non-survivors, compared with survivors. Based on these findings, they speculated that the interaction of inflammatory and anti-inflammatory LMs might have a key role in sepsis^33^. However, there have been no reports regarding the regulation and function of LM dynamics in neonatal sepsis. Here, we clarified the involvement of LMs in the pathogenesis of a preterm sepsis mouse model, and suggest that the protective effect of rhTM in this model might be mediated by LM regulation.

In human studies, rhTM administration was reported to improve the survival rate in DIC associated with sepsis in an adult population^13^, and this has been proven in systematic reviews^34^. Regarding the therapeutic use of rhTM for neonates, its efficacy and safety in neonatal DIC were similar to those of adults^17^. Thus, rhTM might be an option for the treatment of neonatal sepsis.

In this study, administration of 3 mg/kg of rhTM significantly suppressed increases in five LMs at 3 h post-sepsis induction, although these LMs rebounded at 6 h post-sepsis induction. These findings suggest that the protective effect of rhTM is transient and that a sufficient blood concentration cannot be maintained with a dose of 3 mg/kg of rhTM. Thus, a higher dose is needed to maintain the blood rhTM concentration at a sufficient level; accordingly, we examined the effects of a higher dose of rhTM (10 mg/kg). We found that the mortality was significantly increased, such that it was higher than the rate in the Veh-CS-treated septic group. This might be due to the coagulation abnormality caused by an excessive concentration of rhTM. Recently, there have been reports regarding a fusion protein of TM [single-chain variable fragment antibody (scFv)/TM] that targets red blood cells, resulting in extended circulation time and an enhanced TM effect^35,36^. scFv/TM has more potent anti-inflammatory and anticoagulant effects than soluble TM (sTM); thus, scFV/TM shows an efficacy similar to that of sTM at 50-fold lower doses^37^. A single systemic injection of scFv/TM showed remarkably superior outcomes, compared with those of sTM, in animal models of thrombosis and sepsis^38^. In particular, scFv/TM provided both prophylactic and therapeutic protective effects in a mouse model of sepsis, whereas sTM provided inferior prophylactic effects and failed to provide therapeutic effects^37^. Thus, further studies are needed using scFv/TM in our neonatal sepsis model; we speculate that scFV/TM might be able to suppress the inflammatory LM rebound observed at 6 h post-sepsis induction.

This study had some limitations. First, we did not measure the coagulation and fibrinolytic functions. Regarding coagulopathy, previous studies with sepsis patients reported side effects of rhTM based on the presence or absence of bleeding symptoms^17,34^. In this study, we did not observe bleeding, even in neonatal pups treated with a high dose (10.0 mg/kg) of rhTM. However, significant mortality was observed in our rhTM10-CS group, which might have been due to coagulation abnormalities. Thus, a future study should include measurement of clotting ability to elucidate the mechanism of this adverse effect. APC, a coagulation factor produced following rhTM administration, is involved in the cross-talk between blood coagulation and inflammation. APC was reported to exert an anti-inflammatory effect by suppressing the nuclear translocation of NF-κB and inhibiting the production of inflammatory cytokines including TNFα, IL-1β, IL-6, and IL-8^39^. Therefore, the evaluation of coagulation factors, such as APC, is essential to clarify the mechanism of the anti-inflammatory activity of rhTM in preterm sepsis. In addition, in a clinical trial involving the use of recombinant human APC for treatment of children with severe sepsis, an increased risk of bleeding was noted in neonates after treatment^40^. Thus, in preterm infants, the risk of bleeding may increase due to the action of rhTM via APC; future studies should include evaluations of APC, such as the contribution of thrombin-dependent APC pathways, as well as direct quenching of HMGB-1 and other mediators via lectin-like domains.

Second, the timing of rhTM administration in this study was before the induction of sepsis, which was preventative and therefore not therapeutic. Although rhTM is administered via the intravenous route in clinical settings, it is difficult to place a venous catheter in neonatal mice; therefore, we chose subcutaneous administration in this study. Based on the pharmacological kinetic data for rhTM^20^, we subcutaneously administered it at 6 h prior to sepsis induction, which achieved a sufficient blood concentration in adult rats. Based on the above, we think this result reflects the protocol used for clinical rhTM therapy using the intravenous route at the onset of sepsis. However, this study investigated pre-sepsis treatment, rather than post-sepsis treatment. Thus, to determine the therapeutic efficacy of TM, further post-sepsis treatment studies are necessary using large animal models in which an intravenous route can be secured, or using scFv/TM, which has shown protective effects even when administered after the induction of sepsis in an adult mouse model of sepsis.

Third, we have performed bioassays at two very early points to elucidate the acute LM response in this model^41^. However, we could not determine whether the elevation of LMs at 6 h represents the true peak of the response. Thus, to characterise the detailed mechanism by which rhTM influences LM dynamics, further studies are necessary including later time points, such as 9 h or 12 h. In addition, histological evaluation was not performed in this study. Because most Veh-treated controls (71.4%) died within 48 h post-sepsis induction, it was difficult to collect specimens for histological analysis. To further elucidate the pathophysiology, we plan to conduct a study that includes histologic examination using a newly established murine model of sepsis with a prolonged disease course and high survival rate^42^. Although we utilised decapitation for blood collection in our study, this procedure is not ideal. Intracardiac puncture might be desirable in future studies, as it is more ethical and technically feasible.

In conclusion, we report that subcutaneous rhTM administration attenuated sepsis severity in a non-surgical preterm sepsis mouse model. Furthermore, several inflammatory LMs were increased at 3 and 6 h post-sepsis induction, and these increases were suppressed by rhTM pre-treatment only at 3 h post-sepsis induction. Thus, we concluded that LMs might be involved in the pathogenesis of preterm sepsis. scFv/TM might be a promising option to more effectively control post-septic elevation of inflammatory LMs.

## Supplementary information

Supplemental Table.

## Data Availability

The datasets generated during the current study are available from the corresponding author on reasonable request.
