# Supplementary material for: Recombinant human thrombomodulin attenuated sepsis severity in a non-surgical preterm mouse model
Source: Sci Rep. 2020 Jan 15;10:333. doi: 10.1038/s41598-019-57265-2 (PMC6962223; doi:10.1038/s41598-019-57265-2)
Supplement: Supplementary file 1 — Supplemental Table. [file 41598_2019_57265_MOESM1_ESM.docx]

**Supplementary information**

**Recombinant human thrombomodulin attenuated sepsis severity in a non-surgical preterm mouse model**

Mariko Ashina^1^, Kazumichi Fujioka^1,*^, Kosuke Nishida^1^, Saki Okubo^1^, Toshihiko Ikuta^1^, Masakazu Shinohara^2^, and Kazumoto Iijima^1^

^1^Department of Pediatrics, Kobe University Graduate School of Medicine, Kobe, Japan

^2^Division of Epidemiology, Kobe University Graduate School of Medicine, Kobe, Japan

***Corresponding Author:** Kazumichi Fujioka, MD, PhD

Department of Pediatrics

Kobe University Graduate School of Medicine

7-5-2, Kusunoki-cho, Chuo-ku

Kobe, 650-0017, Japan

Tel: (+81)-78-382-6090

Fax: (+81)-78-382-6099

E-mail: [fujiokak@med.kobe-u.ac.jp](mailto:fujiokak@med.kobe-u.ac.jp)

**Supplemental Table S1. LM parameters without significant changes**

| **LM**  **(pg/mg tissue)** | | **Non-septic control** | **3 h post-sepsis induction** | | **6 h post-sepsis induction** | |
| --- | --- | --- | --- | --- | --- | --- |
|  |  | **Veh-Veh**  **(n=4)** | **Veh-CS**  **(n=5)** | **rhTM3-CS**  **(n=6)** | **Veh-CS**  **(n=5)** | **rhTM3-CS**  **(n=5)** |
| DHA | 4-HDHA | 2.0±0.7 | 20.1±7.9 | 6.0±2.5 | 7.3±1.9 | 8.5±3.0 |
|  | 7-HDHA | 0.7±0.3 | 1.3±0.4 | 0.8±0.2 | 1.8±0.5 | 1.7±0.6 |
| AA | PGE_2_ | 41.0±7.9 | 103.3±43.4 | 88.3±19.9 | 57.4±10.7 | 45.8±12.0 |
|  | LTB_4_ | 0.1±0.0 | 0.1±0.0 | 0.2±0.1 | 0.5±0.2 | 0.3±0.1 |
|  | Lipoxin A_4_ | 2.5±0.4 | 5.6±1.4 | 4.5±0.7 | 5.7±1.2 | 3.2±0.9 |
|  | 5,15-diHETE | 1.8±0.5 | 1.4±0.4 | 1.2±0.2 | 2.9±0.8 | 2.3±0.6 |
|  | 5-HETE | 3.6±1.1 | 9.1±3.7 | 5.2±1.3 | 13.5±3.7 | 11.1±4.0 |
|  | 15-HETE | 4.1±0.9 | 14.8±4.8 | 8.7±1.1 | 16.7±5.0 | 10.7±3.3 |

Results are expressed as mean ± SEM.

**Supplemental Table S2.** *Δ***LM parameters without significant changes**

| *Δ* **LM**  **(pg/mg tissue)** | | **3 h post-sepsis induction** | | **6 h post-sepsis induction** | |
| --- | --- | --- | --- | --- | --- |
|  |  | **Veh-CS**  **(n=5)** | **rhTM3-CS**  **(n=6)** | **Veh-CS**  **(n=5)** | **rhTM3-CS**  **(n=5)** |
| EPA | 5-HEPE | 0.2±0.2 | 0.1±0.2 | 1.3±0.4 | 1.9±1.3 |
|  | 12-HEPE | 34.0±12.2 | 8.0±3.8 | 14.3±5.3 | 8.0±3.1 |
|  | 15-HEPE | 0.6±0.3 | 0.3±0.1 | 1.3±0.4 | 1.7±0.8 |
|  | 18-HEPE | 1.5±0.8 | 0.8±0.2 | 2.5±0.7 | 2.7±1.4 |
| DHA | 4-HDHA | 18.1±7.9 | 4.0±2.5 | 5.3±1.9 | 6.5±3.0 |
|  | 7-HDHA | 0.5±0.4 | 0.0±0.2 | 1.0±0.5 | 1.0±0.6 |
|  | 14-HDHA | 15.2±5.0 | 5.1±2.4 | 6.2±2.3 | 6.9±2.2 |
|  | 17-HDHA | 4.7±2.5 | 2.0±1.2 | 6.2±2.0 | 4.1±2.4 |
|  | PD1 | 0.1±0.0 | 0.2±0.0 | 0.1±0.0 | 0.1±0.0 |
| AA | PGE_2_ | 62.3±43.4 | 47.3±19.9 | 16.3±10.7 | 4.8±12.0 |
|  | PGD_2_ | 541.7±150.6 | 358.2±41.5 | 197.2±89.6 | 24.0±40.6 |
|  | LTB_4_ | 0.1±0.0 | 0.1±0.1 | 0.4±0.2 | 0.2±0.1 |
|  | 15deoxy-d12,14 PGJ_2_ | 149.7±31.9 | 98.4±19.7 | 33.1±27.0 | 33.3±22.0 |
|  | PGF_2a_ | 169.7±73.4 | 98.3±15.0 | 118.9±46.2 | 112.5±21.2 |
|  | Lipoxin A_4_ | 3.0±1.4 | 2.0±0.7 | 3.1±1.2 | 0.6±0.9 |
|  | 5,15-diHETE | −0.5±0.4 | −0.6±0.2 | 1.1±0.8 | 0.5±0.5 |
|  | 5-HETE | 5.5±3.7 | 1.6±1.3 | 9.8±3.7 | 7.5±4.0 |
|  | 12-HETE | 152.5±53.5 | 40.8±18.4 | −0.1±3.7 | −2.5±4.0 |
|  | 15-HETE | 10.7±4.8 | 4.6±1.1 | 12.6±5.0 | 6.6±3.3 |

Changes from baseline (non-septic control) levels are abbreviated as *Δ* LM. Results are expressed as mean ± SEM.
